# Controlled Delivery of Insulin-like Growth Factor-1 from Bioactive Glass-Incorporated Alginate-Poloxamer/Silk Fibroin Hydrogels

**DOI:** 10.3390/pharmaceutics12060574

**Published:** 2020-06-20

**Authors:** Qing Min, Xiaofeng Yu, Jiaoyan Liu, Yuchen Zhang, Ying Wan, Jiliang Wu

**Affiliations:** 1 School of Pharmacy, Hubei University of Science and Technology, Xianning 437100, China; baimin0628@hbust.edu.cn (Q.M.); zhangych@hbust.edu.cn (Y.Z.); 2 College of Life Science and Technology, Huazhong University of Science and Technology, Wuhan 430074, China; m201771729@hust.edu.cn (X.Y.); liujiaoyan@hust.edu.cn (J.L.)

**Keywords:** alginate–poloxamer copolymer, silk fibroin, dual network hydrogel, mesoporous bioactive glass, insulin-like growth factor-1

## Abstract

Thermosensitive alginate–poloxamer (ALG–POL) copolymer with an optimal POL content was synthesized, and it was used to combine with silk fibroin (SF) for building ALG–POL/SF hydrogels with dual network structure. Mesoporous bioactive glass (BG) nanoparticles (NPs) with a high level of mesoporosity and large pore size were prepared and they were employed as a vehicle for loading insulin-like growth factor-1 (IGF-1). IGF-1-loaded BG NPs were embedded into ALG–POL/SF hydrogels to achieve the controlled delivery of IGF-1. The resulting IGF-1-loaded BG/ALG–POL/SF gels were found to be injectable with their sol-gel transition near physiological temperature and pH. Rheological measurements showed that BG/ALG–POL/SF gels had their elastic modulus higher than 5kPa with large ratio of elastic modulus to viscous modulus, indicative of their mechanically strong features. The dry BG/ALG–POL/SF gels were seen to be highly porous with well-interconnected pore characteristics. The gels loaded with varied amounts of IGF-1 showed abilities to administer IGF-1 release in approximately linear manners for a few weeks while effectively preserving the bioactivity of encapsulated IGF-1. Results suggest that such constructed BG/ALG–POL/SF gels can function as a promising injectable biomaterial for bone tissue engineering applications.

## 1. Introduction

The extracellular matrix (ECM) is the noncellular component presenting within all tissues and organs, and it usually has three-dimensional porous architecture. It provides not only essential physical scaffolding for the cellular constituents but also initiates crucial processes that are required for tissue morphogenesis, differentiation, and homeostasis [1]. Each tissue has its own ECM with a unique composition and topology, generated during tissue development [2]. Cells interact with biochemical and biophysical cues in their ECM in highly dynamic and reciprocal manners, and such cell–ECM interactions play a critical role in cell behavior mediation, cell function normality and even cell fate decisions, involving quite complicated processes from quiescence to activation and progenitor state to terminal differentiation [1,2]. Accordingly, it is pivotal to harness the interactions between ECM and the resident cells in developing strategies for effectively regenerating tissue or regulating disease [1,2]. To data, many kinds of hard biomaterials have been employed in tissue engineering for various purposes, but their applications in ECM biomimicry have been limited because these hard biomaterials are lack of ability to adequately mimic the structure and properties of ECM in body tissues [3]. Polymer hydrogels, which behave like soft and elastic objects, are usually constructed by physically or chemically crosslinked macromolecules. They contain large amounts of water while having highly porous architecture with tailorable physiochemical properties and easy diffusivity of small molecules. These features make them attractive candidates for mimicking the dynamic ECM [3,4].

Injectable polymer hydrogels having in situ gelling properties under physiological conditions have received a great deal of attention in tissue engineering owing to their two advantageous characteristics [5]. One is that they can be conveniently used to deliver cells, bioactive compounds, and therapeutic drugs alone or in combination by simply mixing these consignments with polymer solutions prior to gelation; and another is that they can be injected to the defect site via minimally invasive surgery followed by formation into solid-like fillers with discretional shapes [5,6]. Nowadays, varied kinds of natural polymers have been commonly used in the form of hydrogels. Among them, alginate has been extensively investigated for its hydrogel applications. Physically crosslinked alginate hydrogels can be easily built by using certain divalent cations, typically calcium ions (Ca^2+^), as a crosslinker. Despite the convenience of preparation, so constructed alginate hydrogels often undergo progressive disintegration in vivo due to the ionic exchange between Ca^2+^ in the gels and monovalent cations (such as Na^+^ and K^+^) coming from the host tissue surrounding the applied gels, which often results in their unstable dimension and uncontrolled properties [7,8]. Another type of physically crosslinked alginate hydrogel was engineered by grafting a type of thermosensitive polymer, poloxamer (Pluronic F127), onto alginate backbone, and the sol-gel transition of alginate–poloxamer (ALG–POL) hydrogels can be trigged by thermosensitive action arisen from the poloxamer component [9]. Despite easy-handled and safe advantages, ALG–POL gels appear to be weak and brittle in nature, and are apt to disintegrate due to its high percentage of Pluronic F127 [10]. Thus, ALG–POL gels are incompetent for certain applications in cartilage or bone tissue engineering where sufficient strength and persistent dimension stability are concomitantly required.

Many studies have revealed that hydrogels with dual or multiple networks could be largely enhanced in their stability and mechanical performance when compared to the single network gel [11]. In spite of the mentioned advantages, multiple network gels are not all suitable for tissue engineering applications as they are usually fabricated via chemical crosslinking, and the involved crosslinking reactions could possibly impair the loaded cells or the host tissue surrounding the applied gel [5,12]. Silk fibroin (SF) is a kind of natural fibrous protein and it can be processed into hydrogels via enzyme-catalyzed crosslinking of amino acid phenolic groups by the aid of H_2_O_2_, and the obtained SF hydrogels show tunable strength and elasticity [13]. In the case of in vivo usage, the applied amount of H_2_O_2_ for crosslinking SF gels has to be controlled at a safe level since the resulting SF gels could be cytotoxic if the applied dose of H_2_O_2_ is higher than certain thresholds [14]. As a result, so prepared SF gels were usually weak [15]. Taking into account the gelable characteristics of ALG–POL and SF through their respectively independent gelling mechanisms, it is feasible to construct a new type of ALG–POL/SF gel with dual network structure while having enhanced performance.

Some growth factors have been proved to be highly effective for promoting bone repair, especially taking advantage of the controlled factor release by way of proper carriers [16]. Among various kinds of growth factors, insulin-like growth factor-1 (IGF-1) is considered to be crucial for longitudinal bone growth, skeletal maturation, and bone mass acquisition not only in the bone growth phase of young individuals but also in the maintenance of bone in adult life [17]. In the situation of bone repair, in addition to promoting cell proliferation and matrix synthesis, the applied IGF-1 at the defect site can also induce the chemotactic migration of osteoblasts to the repair site via local concentration gradients established by factor diffusion [18]. Like many other growth factors, IGF-1 has a short half-life when exposed to the circulatory system [19]. Therefore, when the need arises to maintain sustained release of IGF-1 at the local site in vivo, one of the practical strategies for its administration is to encapsulate IGF-1 into certain vehicles to preserve its activity while managing to modulate its dose and action duration. It is generally realized that directly encapsulating growth factors into hydrogels would result in their burst release because hydrogels commonly have rather porous structures with high water content [7,20]. It has been suggested that release kinetics of growth factors delivered by a hydrogel system could be mediated to varied degrees by encapsulating the growth factor into certain microcarriers such as microspheres (MPs) and nanoparticles (NPs) first, and then, embedding the factor-loaded microcarrier into the hydrogel system [20,21,22].

Bioactive glasses (BGs) have now been widely used as an attractive inorganic biomaterial for bone repair since they have the ability to strongly bond bone tissue via a hydroxycarbonate apatite interface layer with composition and function similar to naturally occurring bone hydroxyapatite [23]. Mesoporous BG microspheres or nanoparticles can also serve as a reservoir for delivering therapeutic drugs or bioactive molecules besides their functions for acting as bone repair material [24]. In addition to regular BG MPs or NPs, many of them have been doped with different kinds of compounds, and their ionic dissolution products are capable of inducing osteogenesis or angiogenesis at the bone defect site, depending on the variety of doped elements [25]. In this context, it would be rational to load IGF-1 into porous BG NPs, and then, to incorporate the IGF-1-loaded BG NPs into above mentioned dual network ALG–POL/SF gels. On the basis of such designed strategy, a multifunctional composite hydrogel system with mechanically strong features and capabilities for administering the release of IGF-1 could be obtained. In this study, an attempt was made to achieve this goal. Some formulated IGF-loaded BG/ALG–POL/SF gels were found to be injectable and mechanically strong, and to have affirmative abilities to control the release of IGF-1 while preserving its bioactivity.

## 2. Materials and Methods

### 2.1. Materials

Sodium alginate (ALG, M_n_: 1.3 × 10^5^ Da), Poloxamer 407 (POL, M_n_: 12,600 Da), 1-ethyl-3-(3-dimethylaminopropyl)-carbodiimide (EDC), horseradish peroxidase (HRP), and *N*-hydroxyl succinimide (NHS) were procured from Aladdin Inc (Shanghai, China). Recombinant human IGF-1 and IGF-1 enzyme-linked immunosorbent assay (ELISA) Kit were purchased from PeproTech Inc (Cranbury, NJ, USA) and R&D systems (Minneapolis, MN, USA), respectively. Other reagents and chemicals were of analytical grade and purchased from Sinopharm Inc (Shanghai, China).

SF was produced using Bombyx Mori cocoons according to the reported method [15]. Cocoons were degummed in a Na_2_CO_3_ solution (0.02 M) at 100 °C for 30 min, and the retrieved silk fibers were rinsed with ultrapure water followed by drying in a ventilated hood. The obtained SF fibers were then dissolved in a LiBr solution (9.3 M) at 60 °C with stirring for 5 h, and the prepared solution was dialyzed against distilled water for 2 days using membrane tubes (MW cutoff: 3500) to remove impurities. The achieved dilute SF solution was further concentrated to varied concentrations by immersing the solution-loaded membrane tubes in a 50% PEG20000 solution, and the concentrated SF solutions were stored at 4 °C for further use.

### 2.2. Synthesis of Alginate-Poloxamer Copolymers

A two-step method was used to synthesize alginate–poloxamer (ALG–POL) copolymers. POL was first modified into monoamine-terminated POL (MATP) following reported methods [26,27], and the obtained MAPT was then grafted onto alginate at a fixed feed mass ratio of alginate to MATP at 1:30 to achieve alginate–poloxamer copolymers. Details for the synthesis of MATP and ALG–POL can be found in the Supplementary Materials.

### 2.3. Preparation of Bioactive Mesoporous Glasses

Two kinds of porous mesoporous BG NPs (named as MBG-1 and MBG-2, respectively) with different pore-sizes were prepared following reported methods. MBG-1 NPs were prepared as follows [28]. One gram of hexadecyl-trimethylammonium bromide was dissolved in an emulsion consisted of 150 mL of H_2_O, 2 mL of aqueous ammonia, 40 mL of ethyl ether, 20 mL of ethanol, and 0.1125 g of calcium nitrate (Ca(NO_3_)_2_·4H_2_O). 600 μL of tetraethyl orthosilicate (TEOS) was then added to the mixture at a molar Ca/Si ratio of 15:85. After stirring at 30 °C for 4 h, the white sediment was collected by filtration, washed with distilled water, and dried in air at 60 °C, and finally, calcined at 550 °C for 5 h. The same method was used to prepare MBG-2 NPs with slight modification [29]. The above prepared emulsion was vigorously stirred at room temperature for 30 min, and then, 600 μL of TEOS was added with vigorous stirring at 30 °C for 4 h. The resulting precipitate was collected and processed in the same way as that applied to MBG-1 NPs. Parameters for these BG NPs are given in Table 1.

Several IGF-1 solutions in PBS (pH 7.4) with varied concentration of 50 ng/mL (low dose), 100 ng/mL (medium dose), and 150 ng/mL (high dose) were first prepared. In a typical preparation process, 1 mL of any IGF-1 solutions was introduced into a vial with inner protein-resistant coating, and 10 mg of blank MBG-1 or MBG-2 NPs was then added. The mixture was allowed to incubate overnight on an orbital shaker at 37 °C. The IGF-1-loaded BG NPs were collected by centrifugation and washed with PBS followed by freeze-drying. The amount of IGF-1 loaded in BG NPs was measured basing on the difference of IGF-1 concentrations in the loading medium before and after soaking BG NPs by using IGF-1 ELISA Kit. Loading efficiency (LE) of BG NPs was calculated by the following equation:

LE(%) = (*M*_0_*/M*_1_) × 100% (1)

where *M*_0_ is the mass of IGF-1 encapsulated inside NPs, and *M*_1_ is the feed mass of IGF-1. Parameters for the IGF-1oaded BG NPs are provided in Table 2.

### 2.4. Preparation of Hydrogels

IGF-1-free composite solutions were prepared using ALG–POL, SF solution and blank BG NPs and they were used to construct blank BG/ALG–POL/SF gels for their compositional and structural optimization in order to save costly IGF-1. Some composite solutions containing IGF-1 were also prepared by directly adding IGF-1 or incorporating IGF-1-loaded BG NPs into the aqueous ALG–POL/SF mixture. These solutions were further processed into gels by incubating them in a water bath at 37 °C. The major parameters for them are summarized in Table 3 and Table 4, respectively.

Gelation time was assessed using the inverted tube testing method. Typically, one of the IGF-1-free composite solutions (2.0 mL) was introduced into a glass vial and it was stirred in an ice/water bath for 5 min before being gelled. Fluidity of the composite solution was checked by regularly inverting the vial, and gelation time was recorded starting from the beginning of vial incubation in the water bath and ending at the point when the solution stopped flowing.

### 2.5. Characterization

Fourier transform infrared (FTIR) spectra of samples were performed on a spectrometer (Vertex 70, Bruker, Ettlingen, Germany). ^1^H NMR spectra were recorded on a NMR spectrometer (Ascend TM 600 MHz, Bruker, Rheinstetten, Germany). The weight percentage of POL in ALG–POL copolymers was determined using an elemental analyzer (Vario EL III, Elementar, Hanau, Germany). The morphology of BG NPs was viewed with a transmission electron microscope (TEM, Tecnai G2, FEI, Hillsboro, OR, USA). Hydrodynamic size and zeta (ζ) potential of BG NPs were measured using a dynamic light scattering (DLS) instrument (Nano-ZS90, Malvern Instruments, Worcestershire, UK). A mass of BG NPs (100–150 mg) were dried for 12 h at 120 °C and degassed for 24 h at 200 °C under vacuum. The volume of nitrogen adsorbed and desorbed at different relative gas pressures was measured using a surface area and pore size analyzer (ASAP 2020 Plus, Micromeritics, Norcross, GA, USA), and it was then utilized to construct adsorption–desorption isotherms. The specific surface area was determined with the Brunauer–Emmett–Teller (BET) method. The pore volume and the mean pore size were derived from the adsorption branches of the isotherms using the Barrett–Joyner–Halanda (BJH) method. The porous cross-section structure of dry gels was examined by scanning electron microscopy (SEM, Quanta 200, FEI, Eindhoven, Netherlands). Dry gel samples with known weight (*W*d, g) were immersed in PBS at 37 °C till they attained equilibrium, and their wet weight (Ws, g) was measured after removal of excess surface water with filter paper. Swelling index (SI) of gels was calculated using the following formula:

SI (g/g) = (*W*_s_ − *W*_d_)/*W*_d_ (2)

### 2.6. Rheological Analysis

Rheological measurements of fluids or hydrogels were carried out using a rheometer (Kinexus Pro KNX2100, Southborough, MA, USA) equipped with a parallel-plate sample holder. The temperature sweep curve was recorded in the range of 25 to 45 °C by heating liquid samples at a rate of 1 °C/min, and the incipient gelling temperature (T_i_) of liquid samples was determined at the intersection point of storage modulus (G′) and loss modulus (G″). Isothermal frequency sweep experiments were conducted using hydrogel discs (10 mm in diameter and ca. 4 mm in height) prepared with a punch, and the obtained data were plotted as a function of modulus versus frequency at predefined strain amplitude of 1%. Shear viscosity of liquid samples was measured at 23 °C in a shear rate range between 0.1/s and 400/s.

### 2.7. Release of IGF-1

In vitro IGF-1 release profiles for IGF-1-loaded MBG-1 and MBG-2 NPs were first tested to find out their difference in release rates. Measurements were conducted using several sets of eppendorf tubes, each filled with 500 μL PBS containing 1% (*w/v*) bovine serum albumin. To each eppendorf tube, 5 mg of IGF-I-loaded MBG-1 or MBG-2 NPs was added, and all sets of tubes were incubated in a shaking water bath at 37 °C and 60 rpm for various periods up to 6 days. At predetermined time intervals, eppendorf tubes were withdrawn by group and they were centrifuged for 5 min at around 1000 g to collect supernatants. The release amount of IGF-I was determined using IGF-1 ELISA kit.

In the case of IGF-1 release from gel samples, some cylindrical gel samples were first produced. Each (0.5 mL) of IGF-1-loaded composite solutions (see Table 4) was filled into a cylindrical mold (diameter: 10 mm) and incubated at 37 °C for 20 min for gel formation. The gel samples were then introduced into different vials filled with 3 mL of PBS, and the vials were vortexed on a shaking table at 37 °C and 60 rpm. At prescribed time points, 1 mL of medium was withdrawn with replenishing the same volume of fresh buffer. The released amount of IGF-1 was measured using IGF-1 ELISA Kit.

### 2.8. Bioactivity of Released IGF-1

The IGF-1’s ability to promote the proliferation of osteoblasts was tested to access the bioactivity of released IGF-1 [30,31,32]. MC3T3-E1 cells (Type Culture Collection of the Chinese Academy of Sciences, Shanghai, China) were used as testing cells. Cells were expanded in DMEM supplemented with 10% fetal bovine serum, 1% penicillin/streptomycin in a 5% CO_2_ humidified atmosphere at 37 °C. The expanded cells were resuspended in PBS for further use.

Cells were cultured in 96-well plates at a density of 5 × 10^4^ cells/well in complete culture medium at 37 °C for 24 h. The cells were serum-starved for 24 h, after which the media was replaced with either serum-free media (denoted as control, 0 ng/mL), serum-free media with free IGF-1 (5 or 50 ng/mL), or serum-free media with released IGF-1 (5 or 50 ng/mL). The cells were cultured for varied durations up to 72 h, and their proliferation was determined by 3-(4,5-dimethyl-2-thiazolyl)-2,5-diphenyl-2-H-tetrazolium bromide (MTT, Dojindo, Japan) essay. Briefly, the media was aspirated from the 96-well plates after the prescribed culture period, and a 20 μL aliquot of MTT stock solution and 200 μL of serum-free medium was added to each well. After being further cultured for 4 h, the media was removed from the wells, and 150 μL of DMSO was added to each well with shaking at 37 °C for 15 min. After that, the optical density (OD) was determined at 590 nm using a microplate reader (PerkinElmer Inc, USA).

Cells were also cultured on the surface of gels for making further comparison. Briefly, two kinds of IGF-1-contained composite solutions with their compositions respectively matching with GEL-2 and GEL-4 gels (see Table 4), 200 μL apiece, were pipetted into wells of 24-well plates, and cultured at 37 °C for gel formation. Subsequently, volume of MC3T3-E1 cell suspension (100 μL) was added to each well (5 × 10^4^ cells/well), and cells were then cultured with serum-free media (500 μL) at 37 °C for varied periods up to 72 h. Cell proliferation was assessed by OD measurement using above described MTT assay. Cell cultured under monolayer condition in serum-free media (0 ng/mL) without exposing to gels were used as control.

### 2.9. Statistical Analysis

Data were presented as mean ± standard deviation. Analysis of the difference between groups was performed using one-way ANOVA. The statistical difference was considered to be significant when the *p*-value was less than 0.05.

## 3. Results and Discussions

### 3.1. Analysis of ALG–POL

**Figure 1:**
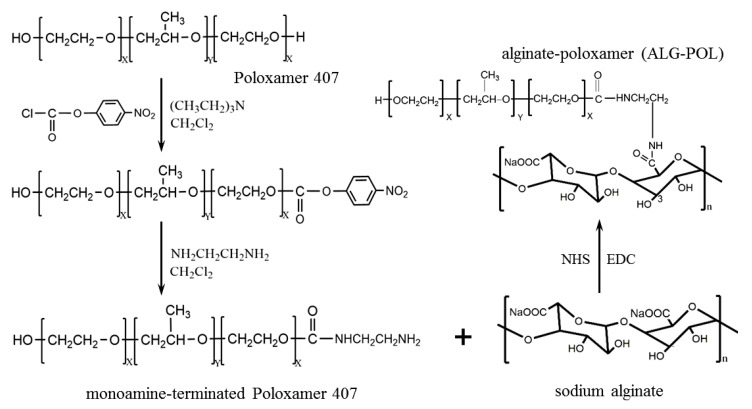
Schematic illustration for synthesis of alginate-poloxamer copolymer.

ALG–POL copolymer was synthesized by grafting MAPT onto the ALG backbone through EDC/NHS chemistry. Figure 1 presents a schematic illustration to show the synthesis route for MAPT and ALG–POL copolymer. A representative NMR spectrum for MAPT is provided in Figure S1 in which the typical peaks respectively assigning to methyl and methylene confirm the successful synthesis of MAPT. The preliminary experimental results indicated that the composition of ALG–POL exerted significant effects on the thermoresponsivity, degradation tolerance, and strength of resulting ALG–POL/SF gels. Accordingly, the proportion of ALG and POL components in ALG–POL copolymer was optimized via orthogonal design method. By changing the ratio of MATP to ALG in a range between 20 and 35 at a step size of 5 during the synthesis of ALG–POL copolymers, one of ALG–POL copolymers was thus screened out by setting the MATP/ALG ratio at 30. The POL content in such optimized ALG–POL copolymer was thus measured to be around 66 wt% via elemental analysis. Figure S2 presents FTIR spectra for POL, ALG and ALG–POL. The spectrum of POL is characterized by three typical bands at 2891 (C–H stretch aliphatic), 1345 (in-plane O-H bend) and 1112 cm^−1^ (C–O stretching). The ALG spectrum shows specific absorbance bands of its COOH stretching at 1610 cm^−1^ and C–O–C stretching at 1305 cm^−1^ [33], respectively. In the spectrum for ALG–POL, the carbonyl absorption band for carboxylate sodium salt originally showing in the ALG spectrum disappeared while a new characteristic amide I band appeared at around 1637 cm^−^^1^, suggesting that amide bonds have formed between ALG and POL [9,33]. FTIR spectra demonstrate that the ALG–POL copolymer has been successfully synthesized.

### 3.2. Parameters for Mesoporous BG Nanoparticles

**Figure 2:**
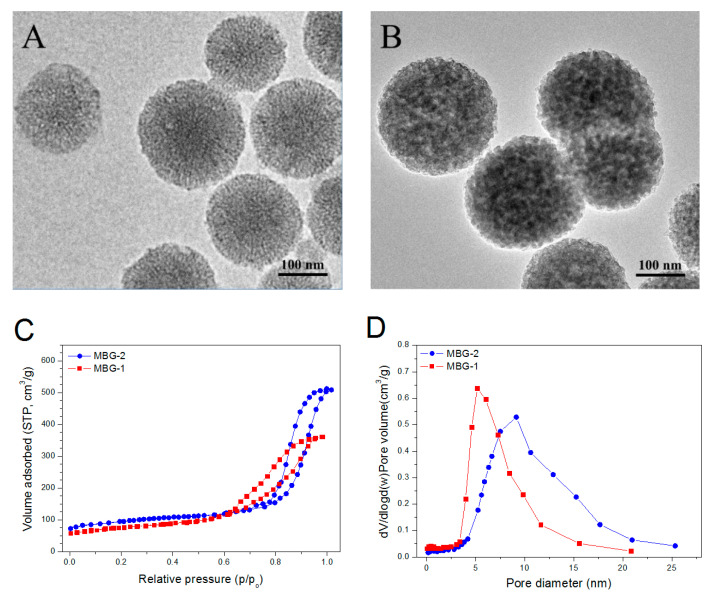
Images of MBG-1 (**A**) and MBG-2 (**B**) nanoparticles (NPs); N_2_ adsorption isotherms (**C**) and pore size distribution (**D**) of NPs.

Two kinds of mesoporous BG NPs were produced under slightly different processing conditions in order to attain certain BG NPs having proper pore-sizes and pore volumes for gaining high LE. Panels A and B in Figure 2 display two typical TEM micrographs for the prepared BG NPs, and these spherical BG NPs were seen to be porous with their size of about 200 nm. A mass of BG NPs were measured for their average particle size and ζ-potential, and relevant data are listed in Table 1. There were significant differences (*p* < 0.05) in the average size and ζ-potential of these BG NPs, signifying that the processing conditions significantly modulated their structure and property even though they were formulated with the same chemical composition [28,29]. Figure 2C displays the recorded N_2_ adsorption–desorption isotherms for MBG-1 and MBG-2 NPs. Two isotherms exhibited their respective hysteresis loops of the desorption branch, indicative of the existence of large pores inside BG NPs [28,29]. In comparison to MBG-1 NPs, the hysteresis loop for MBG-2 NPs was shown to be steeper and its inception turning point was closer to the high pressure end of the N_2_ isotherm, suggesting that MBG-2 NPs have more pores with larger size than MBG-1 NPs. Besides these differences, MBG-2 NPs also quite differed from MBG-1 NPs in pore volume and pore size distribution (Figure 2D). Two sets of BG NPs were measured for their major parameters and the obtained data are summarized in Table 1. It can be seen that the presently developed BG NPs had a high level of pore volume, large average pore size and negative ζ-potential, and thus, they could act as a practical vehicle for the IGF-1 delivery since IGF-1 is somewhat positively charged (isoelectric point, 8.6) at physiological pH and has its molecular weight of about 7.6 kD [34,35].

### 3.3. IGF-1 Release from BG Nanoparticles

Blank MBG-1 and MBG-2 NPs were loaded with IGF-1 under the condition of varying IGF-1 feed amounts, and two sets of IGF-1-loaded BG NPs were thus produced (Table 2). BS*i* (*i* = 1, 2 and 3) sample set was prepared by loading IGF-1 into MBG-1 NPs having smaller average pore size than that for MBG-2 NPs (see Table 1) whereas BL*j* (*j* = 1, 2 and 3) sample set was prepared by loading IGF-1 into MBG-2 NPs. Data in Table 2 reveal that these IGF-1-loaded NPs had similar average particle size (*p* > 0.05) but significantly higher ζ-potential (*p* < 0.001) as compared to their respective blank counterparts (see Table 1). The nearly unchanged average size for IGF-1-loaded NPs shown in Table 2 can be attributed to the very small IGF-1 mass when compared to NPs themselves, whereas the significantly increasing ζ-potential should be ascribed to the slightly positively charged nature of IGF-1 [34]. Table 2 indicates that the IGF-1-loaded NPs in BL*j* (*j* = 1, 2 and 3) set had significantly higher (*p* < 0.05) LE as compared to the counterpart in BS*i* (*i* = 1, 2 and 3) set. These differences are rational because the blank MBG-2 NPs used for preparing BL*j* (*j* = 1, 2 and 3) set have notably higher pore volume and larger pore size when compared to blank MBG-1 NPs used in BS*i* (*i* = 1, 2 and 3) set (see Table 1). Table 2 also shows that the IGF-1 feed amount exerted certain effects on LE, and this kind of effect would become insignificant once the IGF-1 feed amount reached 100 ng/mL or higher. It is worth mentioning that IGF-1 feed amounts were designated as such in order to test the LE for NPs. Actually, the IGF-1 load in these NPs can be effectively regulated by changing the feed amount of IGF-1.

**Figure 3:**
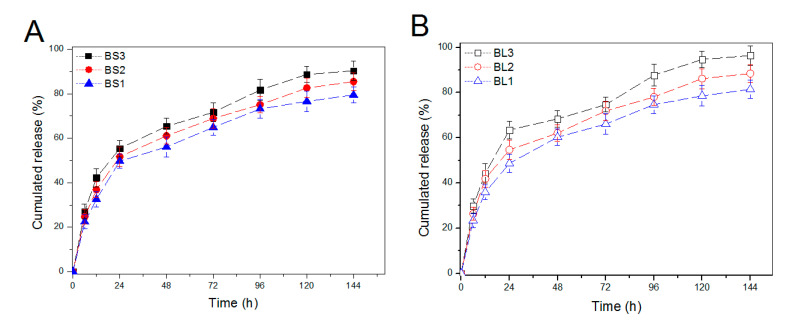
IGF-1 release profiles from 5 mg of BS1, BS2, and BS3 NPs ((**A**), initial IGF-1 load in BS1, BS2 and BS3 was 1.93, 4.61, and 7.27 ng/mg) and BL1, BL2, and BL3 NPs ((**B**), initial IGF-1 load in BL1, BL2, and BL3 was 2.29, 5.72, and 9.21 ng/mg) in 500 μL PBS (see Table 2 for their parameters).

The IGF-1 release patterns from IGF-1-loaded BG NPs are shown in Figure 3A,B. BS1, BS2, and BS3 NPs released around 50% of their initial IGF-1 load on the first day, and after 4-day release, the cumulative IGF-1 release amounted to about 70%. The IGF-1 load in these NPs did not impose any significant impacts on their release profiles. With respect to the cases associated with BL1, BL2, and BL3 NPs, their release profiles looked quite similar to that assigned for BS1, BS2, and BS3 NPs, respectively, with somewhat faster release rates (Figure 3B). Figure 3 verifies that these mesoporous BG NPs themselves are not able to effectively control the release kinetics of IGF-1 on account of their initial burst release features. LE is a key issue that is correlated to the rational use of IGF-1 because of high cost of IGF-1. In consideration of the similar release profiles illustrated in Figure 3 for both MBG-1 and MBG-2 NPs but significantly higher LE detected from MBG-2 NPs (see Table 2), MBG-2 NPs were thus chosen for the follow-up gel preparation.

### 3.4. Rheological Properties of BG/ALG–POL/SF Gels without IGF-1 Load

ALG–POL is a thermoresponsive copolymer and the thermal transition of ALG–POL solutions had strong concentration dependence. A previous study reported that ALG–POL was gelable when its solution concentration reached about 15 wt% or higher [9]. In the present study, the optimally synthesized ALG–POL copolymer was found to have clear sol-gel transition during a rational gelation period when its solution concentration reached 12 wt% or higher. Several optical images are presented in Figure S3 for showing changes of ALG–POL fluids after incubation. It can be noticed that the ALG–POL solution with its concentration of 9 wt% was remained as a fluid even though it was incubated at 37 °C for 60 min, and on the other hand, a 12 wt% ALG–POL solution was able to turn into gel via incubation at the same temperature during 14 min. Results in Figure S3 demonstrate that ALG–POL alone could be thermally gelable at 37 °C when its solution concentration is higher than a certain threshold.

In view of independent gelable mechanisms respectively belonging to ALG–POL and SF components, dual network gels with mechanically strong nature could be constructed by using ALG–POL and SF together. To achieve a ALG–POL/SF gel with required properties, a series of ALG–POL/SF composite solutions having their weight proportions of 4/8, 5/7, 6/6, and 7/5 was formulated for the preparation of blank ALG–POL/SF gels, and the optimal gel was sought out as 5 wt% for ALG–POL and 7 wt% for SF. Based on such designed composition for the ALG–POL/SF gel, blank MBG-2 NPs were embedded into the gel to fabricate three kinds of BG/ALG–POL/SF gels without IGF-1 load and the resulting gels were utilized to evaluate the rheological properties in order to save IGF-1. Major parameters for these blank BG/ALG–POL/SF gels are given in Table 3.

**Figure 4:**
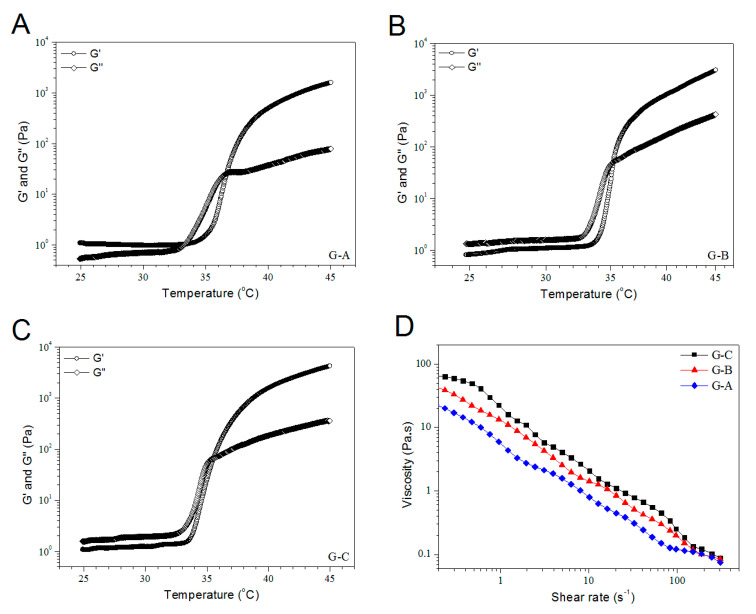
Temperature sweep curves (**A**–**C**) and shear viscosity ((**D**), 23 °C) for BG/ALG–POL/SF gels.

As seen from Table 3, G-A, G-B, and G-C gels had the same matrix and the difference in their composition was the percentage of blank MBG-2 NPs. Panels A, B, and C in Figure 4 elucidate the representative temperature sweep curves of G′ and G″ for different gels, and these gels were seen to respond to the thermal stimulus at different inception temperatures (T_i_). G-A gel had its T_i_ at around 36 °C, whereas G-B and G-C gels showed their T_i_ near 35 °C, connoting that the introduction of BG NPs into the ALG–POL/SF gel has a very limited effect on their gelation temperature. It can be observed from Table 3 that the pH value and T_i_ of these gels were quite close to the physiological pH and temperature, and meanwhile, their gelation time was seen to be rational [5,6], suggesting their applicability under physiological conditions. Figure 4D presents the shear dependence of viscosity for different gels. Their viscosity was shown to be lower than 70 pa.s at 23 °C, and progressively decreased with rising shear rate, indicating their shear-thinning features. Given that the gel injection is usually performed at room temperature, curves graphed in Figure 4D validate that the presently formulated BG/ALG–POL/SF gels have well-defined injectability.

**Figure 5:**
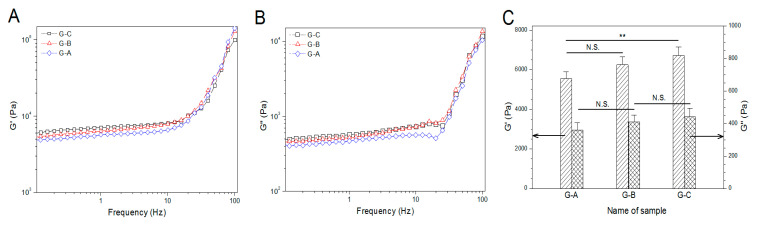
Frequency dependent functions (**A**,**B**) of modulus and average values (**C**) of modulus at 1.0 Hz and 37 °C for BG/ALG–POL/SF gels (**, *p* < 0.001; N.S., no significance).

In principle, magnitude of G′ and G″ of hydrogels in the linear viscoelastic region (LVR) of their frequency sweep spectra together with G′/G″ ratio can be used to assess the gel strength [36]. In general, a strong hydrogel is characterized by high G′, and meanwhile, its G′ should be 1–2 orders of magnitude greater than its G″ [36]. Figure 5A,B show that at a fixed frequency in their respective LVR, for example, 1.0 HZ, three kinds of gels had their G′ of around 5 kPa or higher and their G″ greater than 300 Pa. The incorporation of BG NPs into the ALG–POL/SF gel seemed not to exert marked effects on G′ and G″ of the resulting gels. To make quantitative comparisons, G′ and G″ at 1.0 Hz for these gels were measured, and obtained average values are graphed in Figure 5C. The bar-graphs explicate that these gels had their G′ higher 5.5 kPa with the G′/G″ ratio greater than 15, verifying their mechanically strong features.

### 3.5. Morphological Analysis of Dry Gels

**Figure 6:**
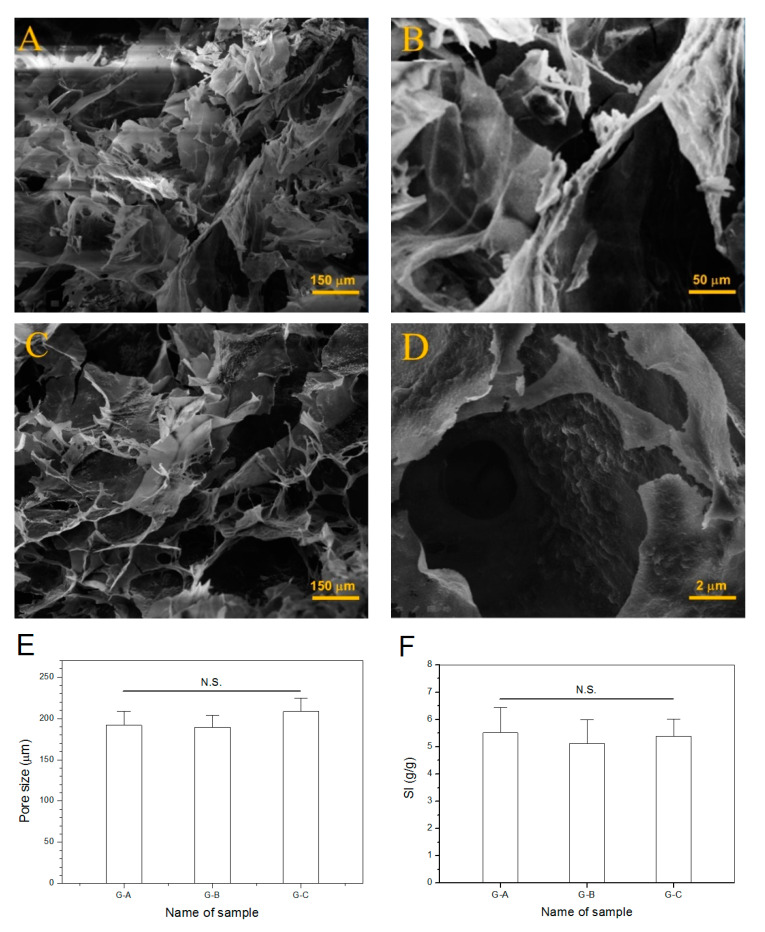
SEM micrographs ((**A**,**B**), G-A gel; (**C**,**D**), G-C gel), average pore-size (**E**) and swelling index (**F**) of BG/ALG–POL/SF dry gels (see Table 3 for parameters of gels; N.S., no significance).

The presently developed BG/ALG–POL/SF gels need to be porous because they are intended for use in bone repair where they will function as injectable materials for housing cells. G-A and G-C gels in Table 3 were selected and their lyophilized samples were examined to see their internal structures. A few SEM images for the dry gels are represented in Figure 6. These images show that dry gels were highly porous and their pore size changed from several tens of microns to more than two hundred microns with pore-interconnected characteristics. With respect to G-C gel, the incorporation of BG NPs did not significantly affect its pore structure when compared with G-A gel. The image with a larger magnification (Figure 6D) displays that the wall of pores in the gel was stuck or attached with many size-varied granules and these granules should be assigned to BG NPs or their aggregates. The average pore size for these dry gels is shown in Figure 6E. The dry gels had large average pore size without significant difference, which is advantageous for bone repair where large pore size and high porosity in the requisite gels are concurrently required.

SI of dry gels has been used as an approximate estimation for their porosity since the channels shaped inside the gels can regulate their swelling and deswelling behavior via water convection [37]. In general, dry gels with open-cell pores and high porosity have high SI and short swelling equilibrium time due to fast water convection [38]. The bar-graph in Figure 6F illustrates that these dry gels had their SI higher than 5, and meanwhile, the composition of the gels seemed not to exert significant impacts on their SI. In addition, it was found that these dry gels reached their respective swelling equilibrium in PBS less than 30 min. The similar SI together with their rapidly swollen features connotes that these gels have similar porosity.

### 3.6. IGF-1 Release of Gels

**Figure 7:**
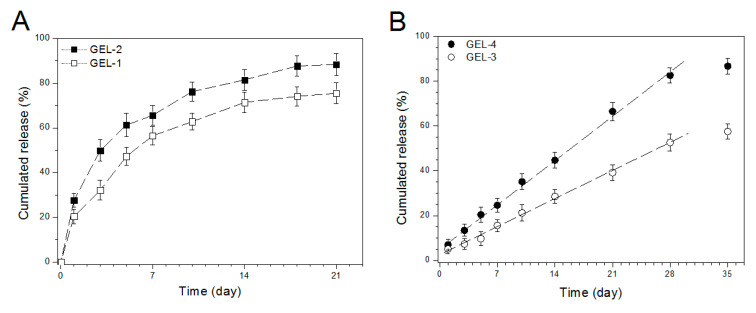
IGF-1 release patterns for gels directly loaded with IGF-1 (**A**) and for IGF-loaded BG/ALG–POL/SF gels (**B**).

The gels were loaded with varied amounts IGF-1 in a designated way as illustrated in Table 4 and they were detected to access their capacity for administration of IGF-1 release. Release profiles for these gels are presented in Figure 7. Curves in Figure 7A exhibit that two kinds of gels directly loading with IGF-1 had fast IGF-1 release in the first few days at varied rates somewhat depending on their initial IGF-1 load, and their IGF-1 release became visibly slower after one-week release with similar release rate in the light of approximately constant distance between the two curves. In marked contrast to this observation, two gels embedded with IGF-1-loaded BG NPs behaved in quite different ways (Figure 7B). IGF-1 load of around 7% or less was released from the gels in the first day, and afterwards, the release patterns followed approximately linear behavior for a few weeks at various release rates. In comparison to the patterns shown in Figure 7A, the significantly reduced initial IGF-1 release and the subsequent release slowdown in Figure 7B can be attributed to the joint contribution of the gel matrix and BG NPs. As denoted in Table 4, GEL-3 and GEL-4 gels were prepared by embedding IGF-1-loaded BG NPs into ALG–POL/SF. In comparison to GEL-1 and GEL-2 gels, IGF-1 molecules in GEL-3 and GEL-4 gels will encounter increasing resistance derived from both BG NPs and gel matrix, which will certainly result in their release slowdown. When the release patterns in Figure 7B are compared each other, it can be observed that IGF-1 content in the gels remarkably affected the release rate. A possible reason could be ascribed to that the higher IGF-1 loading in a gel would form a larger IGF-1 concentration gradient inside the gel, which would force IGF-1 molecules to transport through the gel matrix faster and to reach the media earlier, leading to higher cumulative IGF-1 amount. 

### 3.7. Bioactivity Assessment of IGF-1

**Figure 8:**
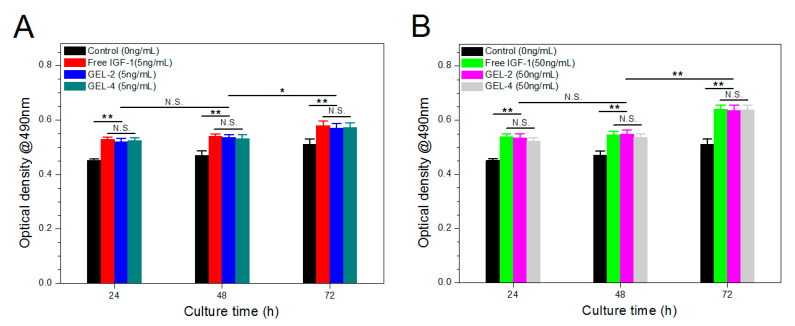
Response of MC3T3-E1 cells to 5 ng/mL (**A**) or 50 ng/mL (**B**) of free or released IGF-1 during various culture periods (*, *p* < 0.05; **, *p* < 0.001; N.S., no significance).

Bioactivity preservation of released IGF-1 is an important issue because it is closely correlated to the biological effects of IGF-1. In this study, MC3T3-E1 cells were used for assessment of IGF-1 bioactivity because IGF-1 is capable of promoting the proliferation of osteoid cells in dose-dependent manners [30,31,32]. To evaluate these gels on the same baseline, GEL-2 and GEL-4 gels were selected taking account of their similar and higher initial IGF-1 load. MC3T3-E1 cells were cultured with equivalent amount of released IGF-1 or free IGF-1 for varied durations up to 72 h and relevant results are elucidated in Figure 8. At a low level of IGF-1 (5 ng/mL), OD values matching with IGF-1-applied cell groups were remarkably higher than that of control group but no significant differences were detected among cell groups that were exposed to free IGF-1 or released IGF-1 as sampling time advanced (Figure 8A). By increasing the applied IGF-1 amount by 10 times (Figure 8B), the variation trend of OD values and their differences looked similar to that detected at the IGF-1 dosage of 5 ng/mL. In addition, by comparing each OD value in Figure 8B with the corresponding one in Figure 8A, IGF-1-dose dependent characteristics can be detected when the culture time reached 72h. These results support that the released IGF-1 was able to promote the proliferation of MC3T3-E1 cells in the way of dose-regulation, confirming that bioactivity of released IGF-1 can be well preserved.

**Figure 9:**
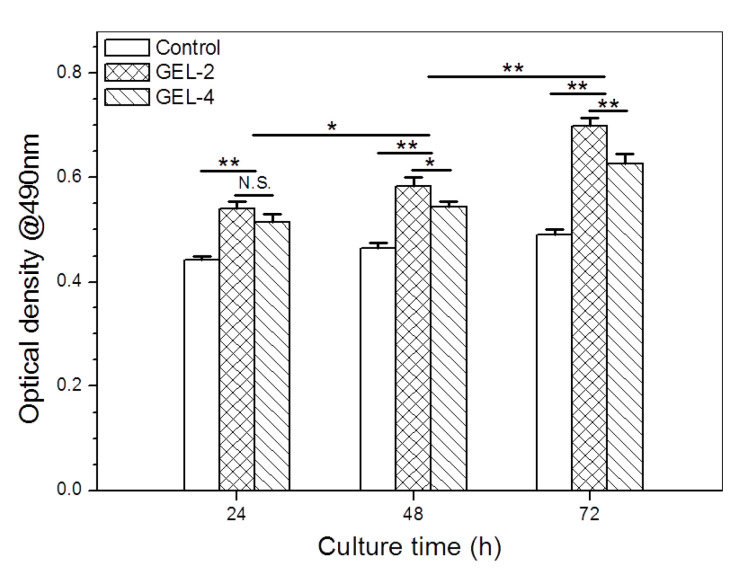
Optical density (OD) values of MC3T3-E1 cells cultured on the surface of gels during various periods (*, *p* < 0.05; **, *p* < 0.001; N.S., no significance).

Besides these tests, MC3T3-E1 cells were also cultured on the surface of GEL-2 and GEL-4 gels for three days to further evaluate their growth, and the measured OD values are depicted in Figure 9. In these cases, the cumulated IGF-1 amount in the culture media would be dynamically altered because GEL-2 and GEL-4 gels would incessantly release IGF-1 at different rates despite their similar initial IGF-1 load. As shown in Figure 7, the cumulative amount of IGF-1 released from GEL-2 gel on the first, second and third days reached around 27, 39, and 50%, respectively; and the corresponding cumulative IGF-1 release from GEL-4 gel was about 7, 10, and 13%. Considering the patterns shown in Figure 7, it can be envisioned that in the current situation, the amount of available IGF-1 in GEL-2 group was significantly higher than that in GEL-4 group in the first three days. It can be seen that OD values measured from two gel groups were markedly higher than that detected from control group during the three sampling days, demonstrating that the released IGF-1 is bioactive and able to promote the growth of MC3T3-E1 cells (Figure 9). When GEL-2 and GEL-4 groups were compared each other, it shows that there was no significant difference in their OD value on the first day, but on the second and third days, OD value measured from GEL-2 group was notably larger than that detected from GEL-4 group. The possible reasons for these observations could be attributed to that (1) cells need a certain period of time to attach to the gels and to undergo recovery growth with low responsiveness to the released IGF-1 on the first day, resulting in insignificant difference in their OD value; and (2) after fully attaching and returning to their normal growth, cells seeded on GEL-2 gel would grow faster than those on GEL-4 gel because GEL-2 gel can release a notable higher IGF-1 amount than GEL-4 gel. These results further confirm that presently devised gels have ability to promote the proliferation of MC3T3-E1 cells.

## 4. Conclusions

Thermosensitive ALG–POL copolymer containing a necessitated percentage of POL was successfully synthesized. Such synthesized ALG–POL was found to be suitable for constructing hydrogels with dual network structure through combining with SF. The optimally obtained ALG–POL/SF gels were injectable at room temperature and mechanically strong with their sol-gel transition near physiological pH and temperature. Embedment of mesoporous BG nanoparticles into ALG–POL/SF gel did not significantly modify the gelation temperature, gelation time and pH of the resulting gels. The interior of the dry gels was seen to be highly porous with well-interconnected pore architecture. Direct incorporation of IGF-1 into ALG–POL/SF gels was inadvisable for administrating IGF-1 release. By embedding IGF-1-loaded BG nanoparticles into ALG–POL/SF gels, the resulting IGF-1-loaded BG/ALG–POL/SF gels showed a confirmative ability to administrate IGF-1 release in an approximately linear manner for a few weeks and their IGF-1 release rate could be effectively regulated by the IGF-1 load in BG nanoparticles. Cell tests confirmed that the released IGF-1 was bioactive as compared with the free IGF-1.

## Figures and Tables

**Table 1:** Parameters for bioactive glass (BG) nanoparticles.

Sample Name	Surface Area (m^2^/g)	Pore Volume (mL/g)	Pore Size (nm)	ζ-Potential (mV)	Particle Size (nm)	PDI
MBG-1	562.4 ± 10.2	0.49 ± 0.02	6.1 ± 0.13	−14.9 ± 0.5	227.2 ± 10.3	0.11
MBG-2	498.3 ± 9.4	0.61 ± 0.03	10.3 ± 0.18	−17.1 ± 0.7	264.7 ± 12.1	0.13

**Table 2:** Parameters for insulin-like growth factor-1 (IGF-1)-loaded BG nanoparticles.

Sample Name	Feed Amount of IGF-1 (ng/mL)	Particle Size (nm)	PDI	ζ-Potential (mV)	LE (%)
BS1 ^(a)^	50	229.1 ± 11.4	0.14	−10.3 ± 0.5	38.6 ± 1.7
BS2	100	230.4 ± 12.6	0.18	−9.1 ± 0.6	46.1 ± 2.1
BS3	150	232.7 ± 10.8	0.19	−8.6 ± 0.3	48.5 ± 2.5
BL1 ^(b)^	50	265.3 ± 11.35	0.17	−10.5 ± 0.5	45.9 ± 2.4
BL2	100	268.2 ± 12.46	0.16	−8.1 ± 0.4	57.2 ± 1.9
BL3	150	267.9 ± 13.14	0.18	−7.2 ± 0.6	61.4 ± 2.3

(a) BS*i* (*i* = 1, 2 and 3) sample set was prepared using blank MBG-1 NPs. (b) BL*j* (*j* = 1, 2 and 3) sample set was prepared using blank MBG-2 NPs.

**Table 3:** Parameters for BG/alginate–poloxamer (ALG–POL)/silk fibroin (SF) hydrogels without factor loading ^(a)^.

Sample Name	SF (wt%)	ALG–POL (wt%)	Blank MBG-2 NPs (wt%) ^(b)^	H_2_O_2_ (µL) ^(c)^	HRP (µL) ^(d)^	pH	Gelation Time (min) ^(d)^	T_i_ (°C)
G-A	7.0	5.0	−	10	10	7.06 ± 0.09	11.75 ± 0.96	36.1 ± 0.42
G-B	7.0	5.0	1.0	10	10	7.13 ± 0.06	10.5 ± 1.29	35.2 ± 0.57
G-C	7.0	5.0	2.0	10	10	7.11 ± 0.07	9.75 ± 0.96	34.6 ± 0.49

(a) The full volume of solutions: 2mL. (b) See Table 1 for their parameters. (c) Concentration of H_2_O_2_: 500 mmol/L. (d) Concentration of HRP: 1000 U/mL. (e) Gelation time was determined by inverting vial every 1 min.

**Table 4:** Parameters for IGF-loaded BG/ALG–POL/SF hydrogels.

Sample Name	SF (wt%)	ALG–POL (wt%)	IGF-1-Loaded MBG-2 NPs (wt%) ^(b)^	H_2_O_2_ (µL) ^(c)^	HRP (µL) ^(d)^	IGF-1 Content in Gel (ng/mL)
GEL-1 ^(a)^	7.0	5.0	−	10	10	154.2 ± 11.27
GEL-2	7.0	5.0	−	10	10	512.9 ± 16.35
GEL-3	7.0	5.0	1.0	10	10	160.3 ± 10.84
GEL-4	7.2	5.0	2.0	10	10	520.7 ± 19.52

(a) GEL-1 and GEL-2 were directly loaded with prescribed amounts of IGF-1 for making comparisons. (b) IGF-1 load inside MBG-2 NPs was regulated by changing the IGF-1feed amount. (c) and (d) See Table 3 for their concentrations.

## Supplementary Materials

The following are available online at https://www.mdpi.com/1999-4923/12/6/574/s1, Figure S1. ^1^H NMR spectra for monoamine-terminated poloxamer (MATP). Figure S2. FTIR spectra for poloxamer (A), alginate (B) and alginate-poloxamer (C). Figure S3. ALG-POL fluids and formed ALG-POL gel after incubation. (Concentration of ALG-POL: 9 wt% (A); and 12 wt% (B)).
