# Supplementary material for: Controlled Delivery of Insulin-like Growth Factor-1 from Bioactive Glass-Incorporated Alginate-Poloxamer/Silk Fibroin Hydrogels
Source: Pharmaceutics. 2020 Jun 20;12(6):574. doi: 10.3390/pharmaceutics12060574 (PMC7355909; doi:10.3390/pharmaceutics12060574)
Supplement: Supplementary file 1 [file pharmaceutics-12-00574-s001.pdf]

pharmaceutics

Supplementary Materials: Controlled Delivery of Insulin-like Growth Factor-1 from Bioactive Glass-Incorporated Alginate-Poloxamer/Silk Fibroin Hydrogels

Qing Min, Xiaofeng Yu, Jiaoyan Liu, Yuchen Zhang, Ying Wan and Jiliang Wu

1. Synthesis of Alginate-Poloxamer Copolymers

Monoamine-terminated POL (MATP) was first synthesized using reported methods [1,2]. In brief, 5 g of POL and 0.2 g of 4-nitrophenyl chloroformate were dissolved in 50 mL of CH_2Cl_2 in presence of triethylamine (0.15 mL), and they were allowed to react at ambient temperature for 4 h to produce an intermediate. The intermediate (5 g) was further reacted with ethylene diamine (1 mL) in methylene chloride (50 mL) for 12 h, and after that, the mixture was extracted with petroleum ether. Such mixture was dialyzed against distilled water using a membrane tube (MWCF: 3500) for 3 days, and lyophilized to achieve MAPT. Subsequently, alginate and MATP (4.5 g) were dissolved in distilled water (50 mL) at a mass ratio of alginate to MATP at 1:30. To this mixture, EDC (0.46 g) and NHS (1.38 g) were added and the reaction was carried out at ambient temperature for 24 h with stirring. Afterwards, the reaction product was dialyzed against deionized water using a membrane tube (MWCF: 12–15k) for 3 days, and freeze-dried to obtain the ALG-POL copolymer.

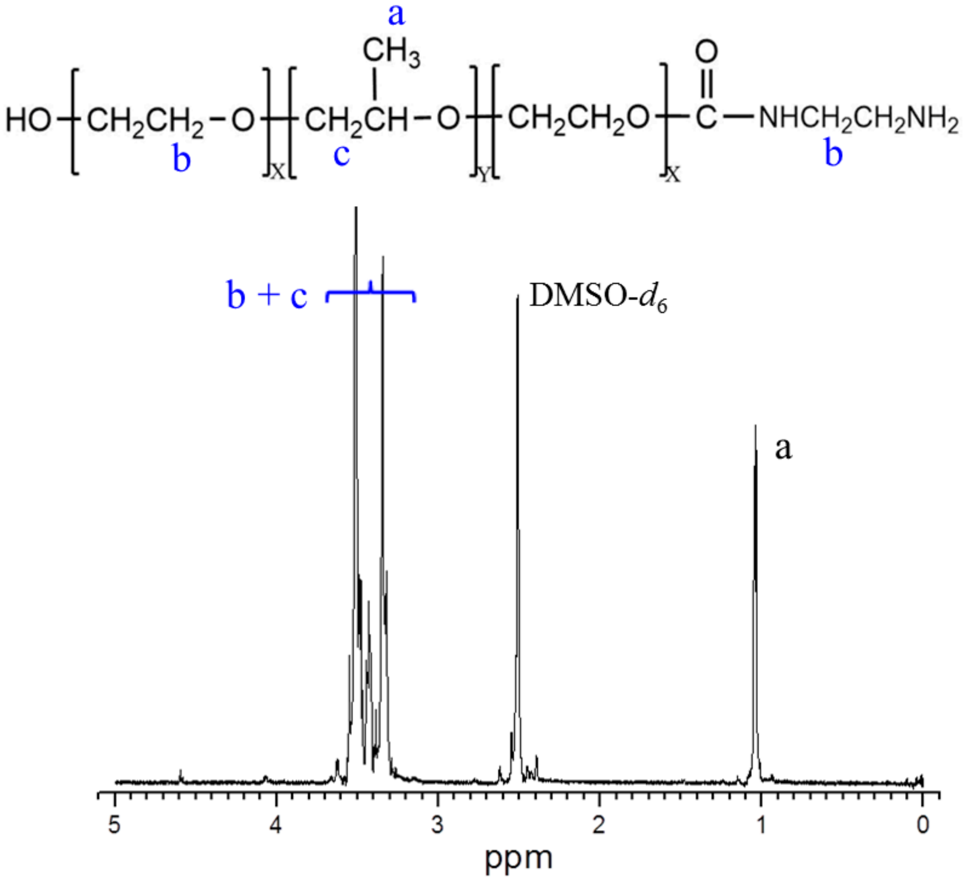

Figure S1. ^{1}H NMR spectra for monoamine-terminated poloxamer (MATP).

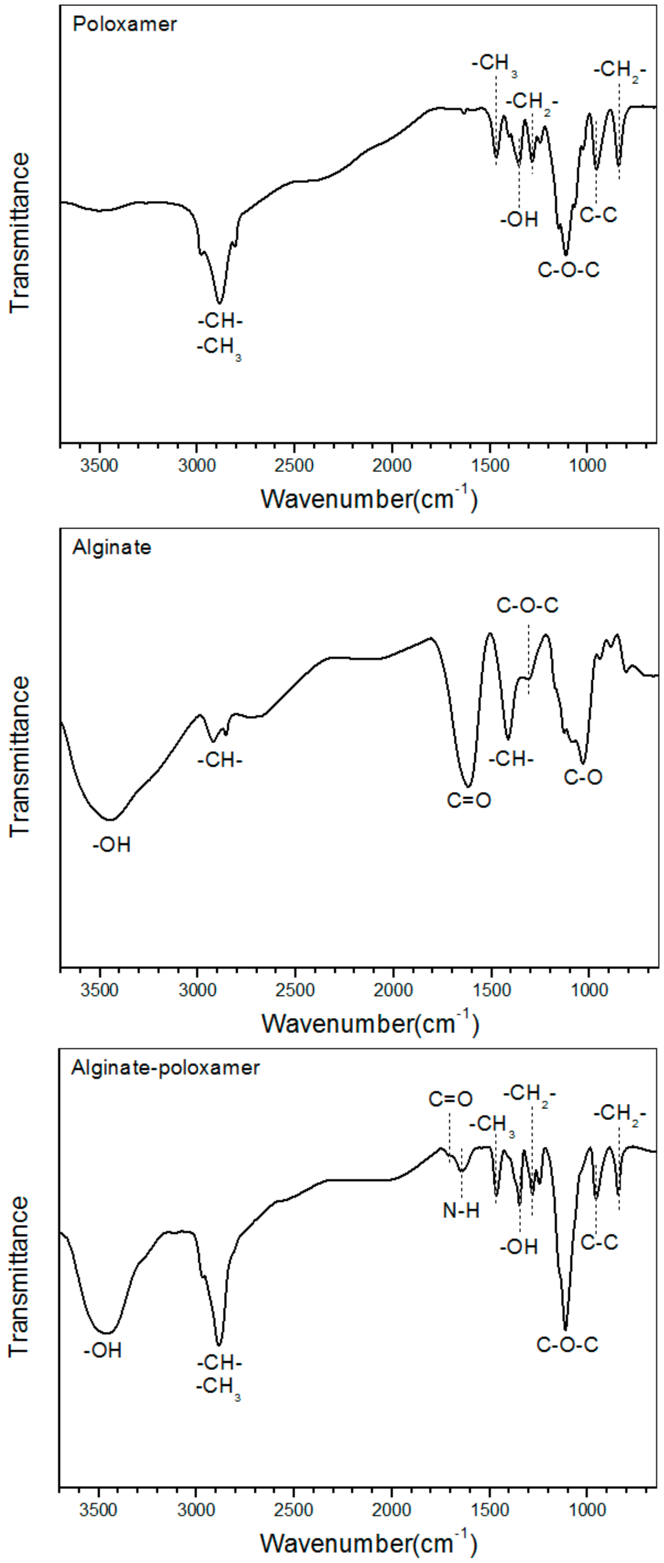

Figure S2. FTIR spectra for poloxamer (**A**), alginate (**B**) and alginate-poloxamer (**C**).

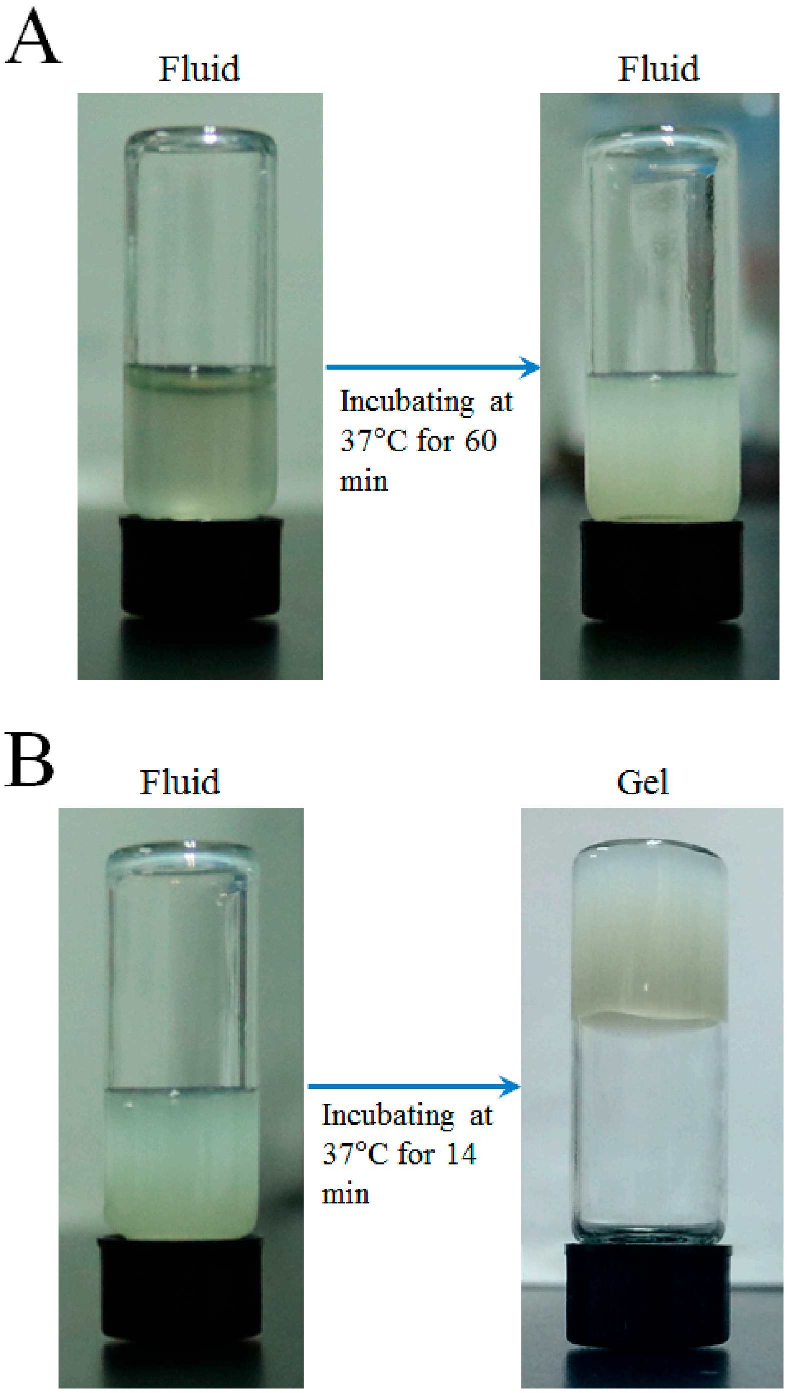

Figure S3. ALG-POL fluids and formed ALG-POL gel after incubation. (Concentration of ALG-POL: 9 wt% (**A**); and 12 wt% (**B**)).

Reference

1. Cho, K.Y.; Chung, T.W.; Kim, B.C.; Kim, M.K.; Lee, J.H.; Wee, W.R.; Cho, C.S. Release of ciprofloxacin from poloxamer-graft-hyaluronic acid hydrogels in vitro. *Int. J. Pharm.* **2003**, *260*, 83–91.
2. Hsu, S.H.; Leu, Y.L.; Hu, J.W.; Fang, J.Y. Physicochemical characterization and drug release of thermosensitive hydrogels composed of a hyaluronic acid/pluronic F127 graft. *Chem. Pharm. Bull.* **2009**, *57*, 453–458.
